# Liesegang Rings in Xanthogranulomatous Pyelonephritis: A Case Report

**DOI:** 10.4061/2010/602523

**Published:** 2010-01-04

**Authors:** Karla Laís Pegas, Maria Isabel Edelweiss, Eduardo Cambruzzi, Cláudio Galleano Zettler

**Affiliations:** ^1^Hospital de Clínicas de Porto Alegre, RS, Brazil; ^2^Universidade Luterana do Brasil, RS, Brazil; ^3^Hospital Nossa Senhora da Conceição, Av. Francisco Trein, 596, Laboratório de Patologia, 2^*∘*^ andar B. Cristo Redentor, Porto Alegre, Rio Grande do Sul, Brazil

## Abstract

Liesegang rings are concentric noncellular lamellar structures, rarely seen in vivo, occurring as a consequence of the accumulation of insoluble products in a colloidal matrix. These characteristic structures are a rare phenomenon usually found in association with cystic or inflammatory lesions and may be mistaken for parasites. The authors examined Liesegang rings from an inflammatory kidney lesion identified previously as a tumoral lesion on computerized tomography. On microscopic evaluation, Liesegang rings can be mistaken for eggs and larvae of parasites, psammoma bodies and calcification. Special stains like PAS, Grocott, von Kossa and Masson's trichrome facilitate the diagnosis.

## 1. Introduction

Liesegang rings are laminated precipitation structures well recognized in the field of chemistry, occurring as a consequence of the rhythmic accumulation of sub- and supersaturation of insoluble products in a colloidal matrix, which precipitate by diffusion resulting in characteristic precipitation rings. They are recognized only rarely in vivo, arising in association with cystic or inflammatory processes. Clinically reported cases have been described most frequently in the kidney and can be mistaken for parasitic infestations. Other reports include paranasal sinus, breast, eye and peritoneum [1–5]. 

 The rings are characterized by periphoeral concentric layers with radial cross-striations that surround an amorphous, central core. They are usually spherical but can vary in shape and size from 5 nm to 820 nm. Concentric laminated morphology can be accentuated with papanicolaou, hematoxylin-eosin, Diff-Quick, Masson's trichrome, von Kossa and Gram stains [6–10]. In the present study, the authors describe Liesegang rings associated with xanthogranulomatous pyelonephritis.

## 2. Case Report

A 51-year-old woman was admitted to the hospital complaining from pain in the left upper quadrant of the abdomen for the last month. The abdominal computerized tomography showed a nodular tumor-like lesion on the upper left pole of the kidney. Partial nephrectomy was performed. The surgical specimen weighted 78,0 g and measured 5,5 × 4,5 × 2,5 cm. On gross examination, there was a poorly circumscribed yellow soft nodule with central degeneration, measuring 3,0 × 2,0 × 2,0 cm.

**Figure 1:**
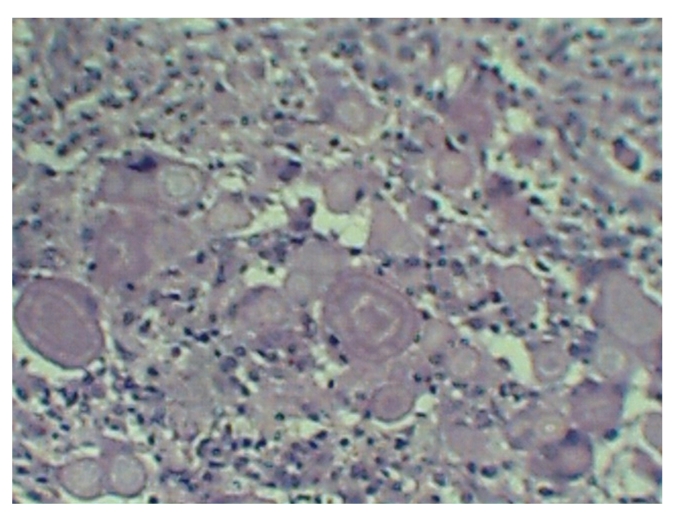
Acellular laminated ring-like structures intermingled with macrophages (Hematoxylin-eosin-100x).

**Figure 2:**
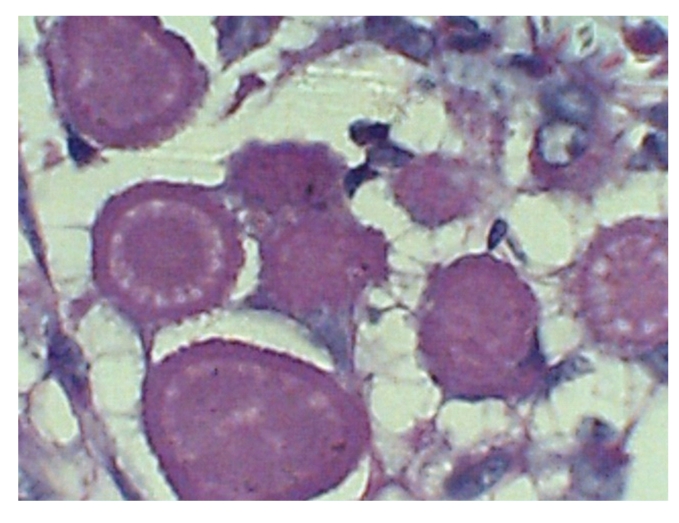
Liesegang rings are positive to PAS stains (400x).

 Microscopically there was a suppurative chronic intersticial nephritis with a lymphoid and neutrophilic infiltrate, central necrotic zone surrounded by foamy macrophages and giant multinucleated cells, tubular atrophy, periglomerular fibrosis and global glomerular sclerosis. The inflammatory process has extended to the medulla and perinephric fat. Next to the central necrotic zone were several spherical laminated rings admixed with macrophages (Figure 1). These ring-like laminated structures resembled psammoma bodies or an ova of a parasite. The rings had an amorphous, central core surrounded by a double layered wall, accentuated with PAS (Figure 2) and negative von Kossa, Grocott and trichrome stains. Under polarized light the rings were nonbirefringent. The findings were diagnostic of xanthogranulomatous pyelonephritis with Liesegang rings.

## 3. Discussion

 Liesegang is an in vitro physico-chemical precipitating process described by the German biochemist Ralph Eduard Liesegang, a German chemist born in Elberfeld who developed also the methods of capillary analysis, a precursor to paper chromatography [11]. These concentric laminated rings are periodic precipitation zones from supersaturated solutions in colloidal systems. They are formed by a process that involves an interplay of diffusion nucleation, flocculation or precipitation, and supersaturation. Examples include Liesegang rings of calcium carbonate in oölitic limestone (in nature), Liesegang rings of silver chromate in gelatin (in vitro), and Liesegang rings of glycoprotein in pulmonary corpora amylacea (in vivo). Though in vivo occurrence is rare, similar physico-chemical factors may be involved in the formation of Liesegang rings, including chemical concentration, matrix medium, temperature, pH and the presence of impurities [1, 7, 8]. 

 Liesegang rings are concentric noncellular structures, occasionally found in the kidney, synovium, conjuctiva, and eyelid. These lesions are spherical to elongated rings formed in cysts, fibrotic tissue, hemorrhagic zones, inflammatory processes and in necrotic areas, and result from a progressive deposition of organic substances, with an unclear pathogenesis. These peculiar ring-like structures range in size from 5 to 820 microns. Most had a double-layer outer wall with equally spaced radial cross-striations and an amorphous central nidus. They are nonpolarizable. In the present study, Liesegang rings were associated with a chronic inflammatory lesion, necrosis and degenerative changes. The characteristic laminated appearance with a double-layered wall, radial cross striations is characteristic and essential for accurate diagnosis [6, 7, 9, 12–14]. 

 The exact composition of the Liesegang rings is not fully understood. Immunohistochemical and histochemical stains for calcium (von Kossa), iron, mucopolysaccharide, amyloid, glycogen, keratin and epithelial membrane antigen are negative. Special stains, radiographic analysis or scanning electron microscopy revealed that some Liesegang rings contained iron, silicon and sulfur. According to Tuur et al. Liesegang rings contain Ca++ besides other inorganic anions, organic polycations and organic polyanions [1]. They have been confused with various parasites, algae, calcification, corpora amylacea, psammoma bodies and the spheroid type of amyloid. Some pathologists have mistaken Liesegang rings for ova, larvae, or adults of the giant kidney worm, *Dioctophyma renale*. The latter is a large blood-red nematode that infects a variety of fish-eating mammals. The adult worms are usually expelled by the uretra and are probably the largest helmint to parasitize humans [6, 7, 10, 12, 15, 16].

## 4. Conclusion

Liesegang rings are a rare histological finding of some inflammatory diseases like xanthogranulomatous pyelonephritis. Pathogenesis remains unclear but it is important to recognize the characteristic features of Liesegang rings to avoid mistaking these with parasites.
